# Barbed channels enhance unidirectional connectivity between neuronal networks cultured on multi electrode arrays

**DOI:** 10.3389/fnins.2015.00412

**Published:** 2015-11-03

**Authors:** Joost le Feber, Wybren Postma, Eddy de Weerd, Marcel Weusthof, Wim L. C. Rutten

**Affiliations:** ^1^Biomedical Signals and Systems, University of TwenteEnschede, Netherlands; ^2^Clinical Neurophysiology, MIRA Institute for Biomedical Technology and Technical Medicine, University of TwenteEnschede, Netherlands; ^3^BIOS Lab-on-a-Chip Group, University of TwenteEnschede, Netherlands

**Keywords:** cortical neurons, multi electrode array, electrophysiology, spontaneous activity, electrical stimulation, stimulus response

## Abstract

Cultured neurons on multi electrode arrays (MEAs) have been widely used to study various aspects of neuronal (network) functioning. A possible drawback of this approach is the lack of structure in these networks. At the single cell level, several solutions have been proposed to enable directed connectivity, and promising results were obtained. At the level of connected sub-populations, a few attempts have been made with promising results. First assessment of the designs' functionality, however, suggested room for further improvement. We designed a two chamber MEA aiming to create a unidirectional connection between the networks in both chambers (“*emitting”* and “*receiving”*). To achieve this unidirectionality, all interconnecting channels contained barbs that hindered axon growth in the opposite direction (from receiving to emitting chamber). Visual inspection showed that axons predominantly grew through the channels in the promoted direction. This observation was confirmed by spontaneous activity recordings. Cross-correlation between the signals from two electrodes inside the channels suggested signal propagation at ≈2 m/s from emitting to receiving chamber. Cross-correlation between the firing patterns in both chambers indicated that most correlated activity was initiated in the emitting chamber, which was also reflected by a significantly lower fraction of partial bursts (i.e., a one-chamber-only burst) in the emitting chamber. Finally, electrical stimulation in the emitting chamber induced a fast response in that chamber, and a slower response in the receiving chamber. Stimulation in the receiving chamber evoked a fast response in that chamber, but no response in the emitting chamber. These results confirm the predominantly unidirectional nature of the connecting channels from emitting to receiving chamber.

## Introduction

In recent years, networks of cultured neurons on multi electrode arrays (MEAs) have received increasing attention to study various aspects of brain functioning (Chiappalone et al., 2008; Eckmann et al., 2008; Pasquale et al., 2008; Vajda et al., 2008; Ide et al., 2010; Ito et al., 2010; Stegenga et al., 2010; Frega et al., 2012; Bakkum et al., 2013; Hofmeijer et al., 2014; le Feber et al., 2014; Stoyanova and le Feber, 2014). Neurons are usually obtained from newborn rats or mice, and are dissociated and plated on the electrode arrays. New axons and dendrites grow out to form randomly connected networks, and cultures become spontaneously active after about a week (van Pelt et al., 2004). Attractive features of this approach include easy access to the neurons, parallel recording from multiple neurons, the possibility to acquire long term recordings (cultures can stay alive up to several months), and large experimental freedom to manipulate the system electrically or pharmacologically. A possible drawback of the current approach is the lack of *in vivo* brain structure. Although many aspects of network functioning of neuronal tissue can be investigated using commercially available MEAs, some applications still require additional structure. Typically, such additional structure involves unidirectional connectivity between two sub-populations, to avoid that the cells in both chambers eventually form one large, oddly shaped network. Recent studies using coupled cultures showed mutual synchronization, where one of the two sub-populations initiated more mutual network bursts than the other (Baruchi et al., 2008; Bisio et al., 2014), suggesting a dominant propagation direction in the connecting axons between the sub-populations. However, the eventual dominant direction of propagation seemed to depend on uncontrolled factors, and could not be predicted. Growth cone guidance has been achieved between specific cell types, or using various forms of surface patterning or scaffolding.

Brewer et al. showed that co-culture of hippocampal dentate gyrus and CA3 neurons have a preferred direction of connectivity between them (Brewer et al., 2013). Although the fraction of axons in either direction differed significantly, selectivity was only ≈60%. Moreover, this natural occurrence of unidirectional connectivity probably strongly depends on the cell types used.

Various attempts have been made to achieve controlled unidirectional coupling between networks of cultured neurons. At the single cell level, several studies showed that surface micro patterning could be used to achieve polarity in the growth direction of the largest neurites, which might be used to create unidirectional connections between groups of neurons (Clark et al., 1993; Stenger et al., 1998; Ruardij et al., 2002; Roth et al., 2012; Scott et al., 2012). At the network level, Pan et al. achieved unidirectional growth by sequential plating of two batches of neurons in two culture chambers connected by micro channels (Pan et al., 2011). By plating the second culture 10 days later than the first one, after axons of the first culture had completely occupied the channels, they aimed to achieve unidirectional coupling. Although seemingly successful, a possible disadvantage of this method is the limited time window for experiments due to this additional delay. Several studies require mature connectivity [>3 weeks old (le Feber et al., 2007)], and cultures often start to deteriorate or detach from the glass surface after ≈4 weeks.

Hattori et al. developed an asymmetric scaffold to promote axon growth in one direction (Hattori et al., 2010), and validated the functionality using electrical stimulation on one side while recording calcium signals at the other side. Their device was shown to favor axon growth in the desired direction, but unidirectionality was far from perfect. Peyrin et al. developed funnel shaped micro channels to create “axonal diodes” (Peyrin et al., 2011). They used staining techniques and calcium imaging to show that many axons grew through the channels in the desired direction, and that oscillatory calcium signals that arose in the “emitting” chamber were transmitted to the “receiving” chamber. They did not electrophysiologically characterize the direction of the connections between the chambers, and their study did not reveal to what extent signal propagation was blocked in the opposite direction. In a later study, they showed that their channel design indeed hampered axon growth in the undesired direction, as demonstrated by a lacking stimulus response in one third of experiments, or by a larger response latency in two thirds of experiments (Renault et al., 2015). It remains difficult, however, to distil to what extent unidirectionality is on average achieved following that approach.

The calcium signaling readouts in these studies did not allow for temporal characterization of the connections, due to the inherently slow nature of calcium signals (Helmchen et al., 1996). Furthermore, these pioneering studies did not investigate if and how the unidirectional character of connectivity between the chambers might be further improved with channels that contain multiple funnels.

Here, we constructed and validated a custom made MEA with two culture chambers (“*emitting*” and “*receiving”* chamber) for simultaneous plating, and interconnecting channels designed to support axon growth in one direction only. The channels contain barbs, creating multiple “funnels” inside each channel, aiming to further improve unidirectionality, while minimizing the time needed to grow through the channels in the desired direction. We electrophysiologically characterized the functionality of the channels, and determined several measures to assess probability of activity propagation in either direction, as well as propagation latency.

## Methods

We designed a device that consisted of two chambers, connected by barbed channels, which we will refer to as double chamber multi electrode array (DCMEA). The barbs in the channels pointed in the desired direction of action potential propagation, aiming to impede axon growth in the opposite direction. Their functionality will be evaluated in this paper. The electrode design in the chambers was optimized to create one target culture, receiving input from the other culture, and one auxiliary culture, providing this input. We will refer to these cultures (chambers) as receiving and emitting chamber, respectively.

We aimed to build DCMEAs to meet with the following requirements: Top and bottom of culture chambers and the channels should be optically transparent for microscopic inspection. Each chamber should have sufficient volume to enable continuous recording for at least 48 h without the need to refresh the growth medium. There should be enough channels to enable significant connectivity between the chambers; these channels should be as short as possible, but long enough to impede axon growth in one direction. The DCMEAs should have enough electrodes in the receiving chamber to characterize the behavior of the receiving network, whereas there should be enough electrodes in the emitting chamber to at least enable successful stimulation. Finally, the DCMEAs should be compatible with Multi Channel Systems (MCS) hardware.

### Construction of the DCMEAs

DCMEAs were composed of two separate structures: a glass bottom layer, containing the electrodes and leads, and a top layer containing the wells and channels.

#### Bottom layer

**Figure 1:**
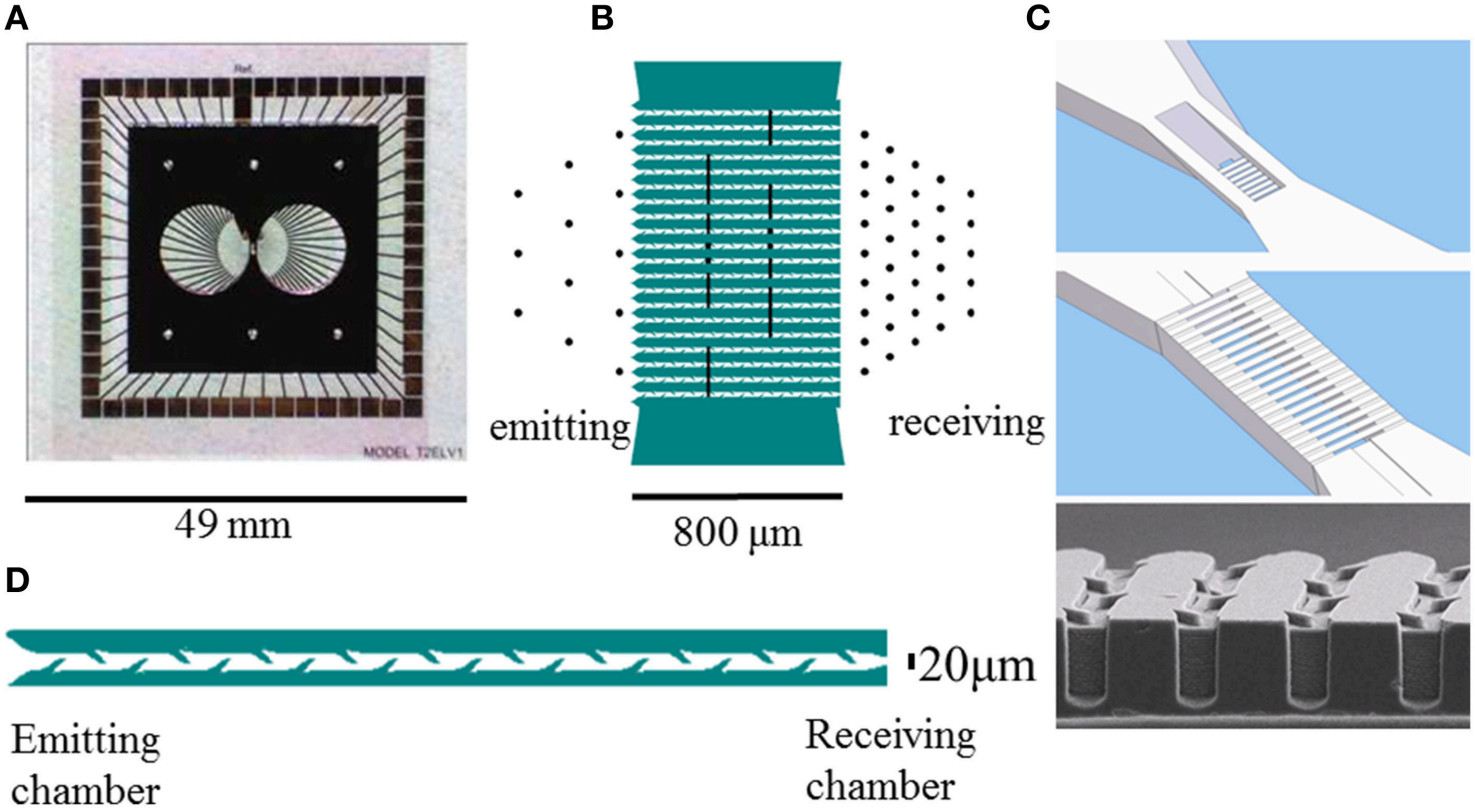
**Layout of the culturing chambers, channels, and electrodes of glass-silicon DCMEAs**. **(A)** shows the complete DCMEA, **(B)** depicts the layout of electrodes (black lines and dots, Ø: 30 μm) and channels (green). **(C)** shows a top and bottom view of the connecting channels. The barbed channels are 5 μm high at the entrance and exit, and the central part of the channels has no ceiling to enable visual access. Total channel length: 800 μm, channels, and walls running from lower left to upper right are indicated in **(B)** in white and green, respectively. Bottom image shows an electron microscopy image of the barbs in the channels. **(D)** Close-up of a channel (10 barbs per side, length:800 μm, width: 20 μm, all dimensions are to scale). Glass PDMS DCMEAs had a similar layout, but the channels were shorter (540 μm, five barbs per side).

We designed the bottom layer to have electrodes in both chambers and in the connecting channels. While recording and electrical stimulation is possible in both chambers, the emitting chamber was optimized for stimulation and the receiving chamber for recording, as reflected by the different numbers of electrodes in each chamber. Electrode spacing was 225 μm in the emitting chamber, and 115 μm in the receiving chamber. Figure 1B shows the electrode layout, electrodes are represented by black dots and vertical lines. All channels contained at least one electrode. The total number of electrodes was limited to 59. Since we already used 35 electrodes to emphasize on characterization of activity in the receiving chamber and 12 in the emitting chamber (see design requirements above), some channels had shared electrodes (e.g., bottom four channels in Figure 1B). To observe signal propagation in the central channels, these nine channels had two electrodes, separated by 240 μm.

First, we etched 250 nm deep patterns into a 500 μm thick four inch pyrex glass wafer to create cavities for the electrodes and leads. Next, an intermediate titanium layer of 200 nm was sputtered on the substrate to form the electrode leads. Two layers of gold, respectively 50 and 400 nm thick (width tapering from 30 to 28 μm) were deposited by sputtering to create the electrodes. Additionally, one large reference electrode was created, such that it was in contact with both chambers. It can be seen in Figure 1A, as the large lead from the top row of contact pads (in the middle), indicated by “ref.” The reference electrode's far ends can be seen in both chambers, right below and slightly to the right of the lead. For electrical insulation a 400 nm layer of silicon nitride was deposited on the pyrex wafer, using Low-pressure Chemical Vapor Deposition (LPCVD). Electrode contact areas were opened by etching through the nitride layer in a reactive ion etcher using CHF_3_. Finally, 49 × 49 mm square areas were cut out of the wafers to fit into an MCS amplifier rack. The position of the reference electrode, the total size of the glass layer, and the positioning of contact pads were tuned to MCS hardware, but might be adjusted to fit other equipment as well.

#### Top layer

A top layer, containing the structures of the chambers and channels was mounted on top of the glass layer. This top layer was constructed in silicon or polydimethylsiloxaan (PDMS). All channels were 20 μm wide, 5 μm high, and contained barbs that locally narrowed the channel width to ≈10μm, as shown in Figure 1D. This height prevented the migration of somata into the channels, and the effective channel width between 10 μm (next to barbs) and 20 μm was shown to maximize successful ingrowth of neurites (Wieringa et al., 2010a; Peyrin et al., 2011). The distance between two neighboring barbs at one side of a channel was 80 μm.

##### Silicon top layer

The silicon top layer was fabricated using a 500 μm thick SOI (silicon on insulator) wafer, which contained a buried insulating layer (0.5 μm SiO_2_), 50 μm below the surface. This enabled us to etch the entrances of the channels on both sides (5 μm deep), the central part of the channels (50 μm deep; 20 μm wide), and the bottom of the chambers (50 μm deep) from the shallow side, and to completely open the chambers and the area above the central part of the channels from the other side without destroying the fragile channel structures. Figure 1C shows a top and bottom view of the silicon layer channel design, as well as an EM photograph. The silicon layer also contained channels for perfusion of the chambers, to facilitate continuous medium refreshment or drug administration. We did not yet use this option, in all experiments described here, the perfusion channels were closed manually with PDMS.

We used anodic bonding to obtain an hermetic seal between the silicon top layer to the glass bottom layer (Wallis and Pomerantz, 1969). To enable proper bonding, the insulating silicon nitride layer was removed from the intended contact spots, but not from surface of the chambers and channels. The two contacted wafers were heated to 250–400°C to mobilize the ions while a voltage of 1000–1500 V was applied. The high voltage created an electric field that pulled the wafer surfaces into intimate contact and fused the wafers together.

We added a PDMS collar of circa 5 mm height to increase the chamber volume of the DCMEAs to ≈350μL. With this chamber volume, two medium refreshments per week were sufficient to keep cultures healthy and active. In future experiments with continuously perfused cultures, this collar will become obsolete. We used a single collar around both culture chambers and the connecting channels. This collar provided good visual access to chambers and channels, but did not support different cell types in the chambers or individual chemical manipulation of either culture. Additionally, it blocked the inlets of the perfusion channels, leaving only a single opening at the top. Finally, the DCMEA was sealed with a glass lid on top of the PDMS collar. We did not experience any difficulty in producing PDMS that was flat enough on both sides to enable good bonding between silicon (at the bottom), glass (at the top), and the intermediate PDMS collar.

##### PDMS top layer

To construct the structures with chambers and channels in PDMS, we used a silicon mold with the same chamber and channel layout as the silicon top layers, but with shorter channels (540 μm, 5 barbs on each side). The PDMS structures had no perfusion channels. The PDMS was ≈5 mm thick, creating chambers of ≈350 μL. The channels of the PDMS top structures had the same dimensions as those in the silicon structures, but the PDMS above the central part of the channels was left intact (in contrast to the opening in the silicon above the central channel area). This somewhat hampered visibility of axons inside the channels. PDMS easily adhered to the glass, and was positioned under an inverted microscope before sterilization.

### Validation of unidirectional connectivity

To validate the functionality of the DCMEAs and the unidirectionality of the channels, we cultured cortical neurons in both chambers, and used visual inspection, electrophysiological recordings, and electrical stimulation to determine the direction of axon growth in the channels.

#### Cell culture

We obtained cortical cells from newborn Wistar rats on post-natal day 1. All surgical and experimental procedures complied with Dutch and European laws and guidelines and were approved by the Utrecht University Animal Experiments Review Committee. After trypsin treatment, cells were dissociated by trituration. Suspensions of dissociated cells contained a random mixture of cortical neuron types, including ~10–25% inhibitory and 75–90% excitatory neurons (Marom and Shahaf, 2002).

About 50,000–100,000 dissociated neurons were plated in both chambers, which were precoated with poly ethylene imine (PEI). This procedure resulted in an initial cell density of approximately 5000 cells per mm^2^. With aging, cell densities gradually decreased to ≈2500 cells/mm^2^, as estimated from countings. Both culture chambers were filled with ≈350 μL R12 medium (Romijn et al., 1984), a serum-free medium that impeded wild proliferation of glial cells. DCMEAs were stored in an incubator, under standard conditions of 37°C, 100% humidity, and 5% CO_2_ in air. For recordings, we firmly sealed the culture chambers with a glass coverslip, and placed the cultures in a measurement setup outside the incubator. This approach enabled recording from healthy cultures during at least 3 h (le Feber et al., 2007; Stegenga et al., 2008), which was sufficient in the current study. For details about the recording setup, see Stegenga et al. (2008). After experiments, cultures were returned to the incubator. Medium was refreshed two times a week. When activity could no longer be measured in one of the chambers, or both, we peeled off the PDMS top layer and thoroughly rinsed the glass and the PDMS layer in demineralized water after a 30 min exposure to BioTex. Both were sterilized before reuse (dry sterilization at 160°C for 3 h).

#### Recordings

All DCMEAs were connected to commercially available hardware (MultiChannelSystems) to obtain electrophysiological recordings. Recordings began after an accommodation period of at least 20 min. We used the MC1060BC preamplifier and FA60s filter amplifier (MultiChannelSystems GmbH, Reutlingen, Germany). Amplification was 1000 times in a frequency range from 100 to 6000 Hz. A 6024E data-acquisition card (National Instruments, Austin, TX) was used to record all 60 channels at a sample frequency f_s_ = 16 kHz. During the experiments, the temperature was controlled at 36°C, using a TC01 (MultiChannelSystems) temperature controller. Electrical stimulation was accommodated by an STG1002 stimulus generator (MultiChannelSystems).

#### Visual inspection of axons inside channels

We stained axons with cell tracker green (5-Chloromethylfluorescein Diacetate; Invitrogen) and counted the number of barbs that the axons in the channels passed in either direction, at different ages. As long as axons did not reach the other chamber, it was often possible to determine from which chamber the axons originated.

#### Spontaneous activity

Cultured cortical neurons usually become spontaneously active after about a week, and develop firing patterns that contain periods of seemingly uncorrelated firing, intermingled with synchronous network bursts, periods of highly synchronized firing at many electrodes (van Pelt et al., 2004). We used spontaneous activity to infer connectivity between the chambers. **Figure 3** shows an example of recorded spontaneous activity in both chambers and in the channels.

Several channels contained two electrodes, as shown in Figure 1B. Note that most electrodes covered more than one channel, which introduces some uncertainty in the location where activity originated. In addition, it is possible that two electrodes in one channel pick up activity from different axons. We cross-correlated the signals (expressed as point processes) in those channels where both electrodes picked up activity, to determine the latency with maximum cross correlation. A clear peak in the cross-correlation curve at short latency indicated that both electrodes probably picked up activity from the same axon in the same channel. This latency could be either positive or negative and was indicative of the direction of action potential propagation.

Secondly, we calculated chamber wide firing rates (CWFRs) as the summed activity recorded at all electrodes of that chamber per 5 ms bins. We cross-correlated CWFRs (predominantly burst envelopes) of both chambers (see **Figure 3C**) to determine the latency with maximum correlation. Assuming that uncorrelated spikes or bursts (i.e., partial bursts, see below) don't contribute to the mean total cross-correlation, this measure gives insight into the temporal order of bursts, and may thus indicate a certain propagation direction.

Finally, we applied a burst detection algorithm to all activity recorded from the emitting chamber, the receiving chamber, or from the channels. We adapted the algorithm by Stegenga et al. (2008), which consisted of two steps. First the number of active electrodes was determined (at least one spike per 10 s). Then, the array wide number of action potentials per 10 ms bins were counted (sum of all electrodes). When this number exceeded a threshold of two times the number of active electrodes, it was considered to be a burst. For both chambers, we calculated the fraction of partial bursts. Partial bursts were defined as bursts that occurred only in that chamber, without a coinciding burst in the other chamber. Bursts duration in cortical cultures older than 20 days is typically less than 200 ms (Wagenaar et al., 2006). We set a (conservative) threshold for bursts in both chambers to coincide at two times that duration. Thus, bursts in both chambers coincided whenever the burst in the second chamber reached maximum firing intensity within 400 ms after the first. Assuming that bursts may be intrinsic or ignited by a burst in the other chamber and then propagated through the channels, this fraction is inversely related to the probability that a burst is propagated to the other chamber. Statistical differences between the fractions of partial bursts were assessed by paired *t*-tests, with significance threshold at *p* = 0.05.

#### Stimulus responses

We electrically stimulated all electrodes at a low frequency (0.2 Hz), and an amplitude that showed clear responses at the other electrodes in the same chamber (biphasic pulses, 200 μs per phase, amplitude: 500–900 mV or 16–30 μA). We calculated post-stimulus time histograms (PSTHs) in both chambers as the summed activity recorded at all electrodes of that chamber per 5 ms bin during the first 200 ms after the stimulus. Besides stimulus induced activity, also spontaneous activity was recorded that sometimes included spontaneous bursts in the 200 ms interval after a stimulus. These spontaneous bursts were not time locked to the stimulus, but might still dominate the estimated PSTH if the burst was relatively large. To avoid this influence, all electrodes were stimulated 10 times, and we included only responses to those stimulation electrodes with PSTHs showing a peak at the same latency after multiple stimuli. We calculated the summed PSTH of all stimulations at a certain electrode *j*, $\sum_{i=1}^{10}{PSTH}_{i,\;j}$, and excluded all responses that were based on a single stimulus, as indicated by max{$\sum_{i=1}^{10}{PSTH}_{i,\;j}$} = max{*PSTH*_*i, j*_}. Here *i* indicates the ordinal number of a stimulation at electrode *j*. This approach ignored outlying PSTHs that possibly resulted from a spontaneous network burst shortly after a stimulus. Average PSTHs were considered to show a significant stimulus response if the 95% confidence interval (mean ± 1.96 × SEM) did not include zero in at least two subsequent time bins.

## Results

We plated 20 times on glass-silicon DCMEAs (each time with cultures in both chambers), and 20 times on glass-PDMS DCMEAs. Remarkably, we were able to record activity in most cultures in glass-PDMS DCMEAs, but never in cultures on the glass-silicon DCMEAs. Still, cultures in glass-silicon DCMEAs appeared healthy and growing upon visual inspection. Cultures in glass-silicon DCMEAs could be used for visual validation of the direction of axon growth inside the channels, but electrophysiological data were obtained only from the glass-PDMS DCMEAs. To facilitate comparison of visual and electrophysiological validation all visual inspection was also done in glass-PDMS DCMEAs. All cultures used for electrophysiological recording were at least 19 days old (range: 19–36 days *in vitro*). At this age, cortical cultures have reached a mature state of stable activity (van Pelt et al., 2004; Stegenga et al., 2008) and connectivity (le Feber et al., 2007).

### Visual inspection of axons inside channels

**Figure 2:**
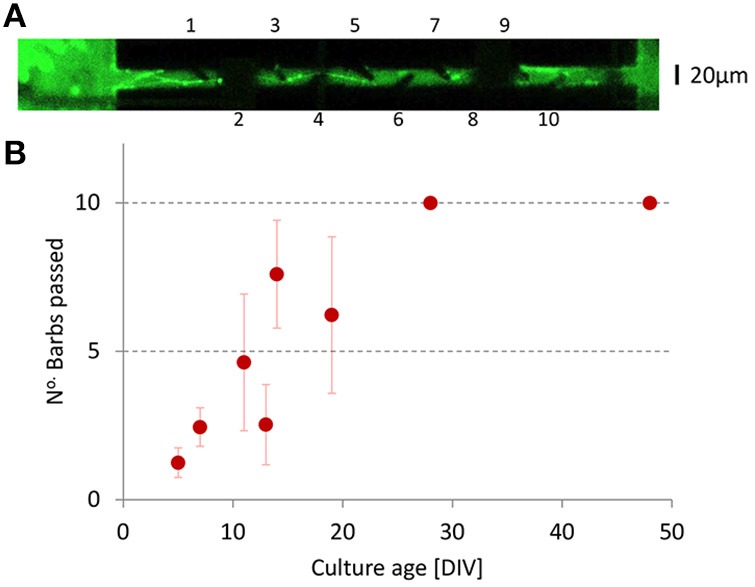
**Axon growth inside the channels. (A)** Neurons and axons were stained with Cell Tracker Green to visualize the axons inside a channel. The image was slightly overexposed to better show the barbs. Protrusion was measured by the number of barbs that the axons passed (barb numbering indicated). In this example, the axon has passed the first five barbs. The channel electrodes obscured the view at two locations (dark areas around barbs 2 and 8). Near barb 10 some ingrowth in the opposite direction and some debris are visible. **(B)** The number of barbs passed in the foreseen direction (left to right) as a function of age. We never saw axons pass more than two barbs in the opposite direction.

In total we labeled seven different cultures with cell tracker green (12 μM, Invitrogen). Figure 2A shows an example. Occasionally, it was possible to follow axons that originated from either chamber into the channels, and to count the number of barbs that they passed. Often, however, it appeared difficult to follow the axons because they disappeared from the focal plane, or because visibility was obscured by light interference patterns near the channel walls or by debris from earlier platings. Axon through growth from emitting to receiving chamber increased with time (see Figure 2B), and axons reached the other end of the channel in ≈20 days. We also saw axons growing in the opposite direction, but these never passed more than two barbs.

### Spontaneous activity

In six experiments we were able to record action potentials at two electrodes within a channel, in 14 channels in total. Five channels (35% of the recorded channels) showed maximum cross correlation at positive latencies (emitting side leading), 1 channel (7%) at a negative latency (receiving side leading), and in the other channels maximum cross correlation was reached at zero lag(*n* = 4), or no clear maximum was found (*n* = 4). Overall mean latency at maximum cross correlation was 0.12 ms, suggesting a propagation speed of 2 ± 1.7 m/s from emitting to receiving chamber.

**Figure 3:**
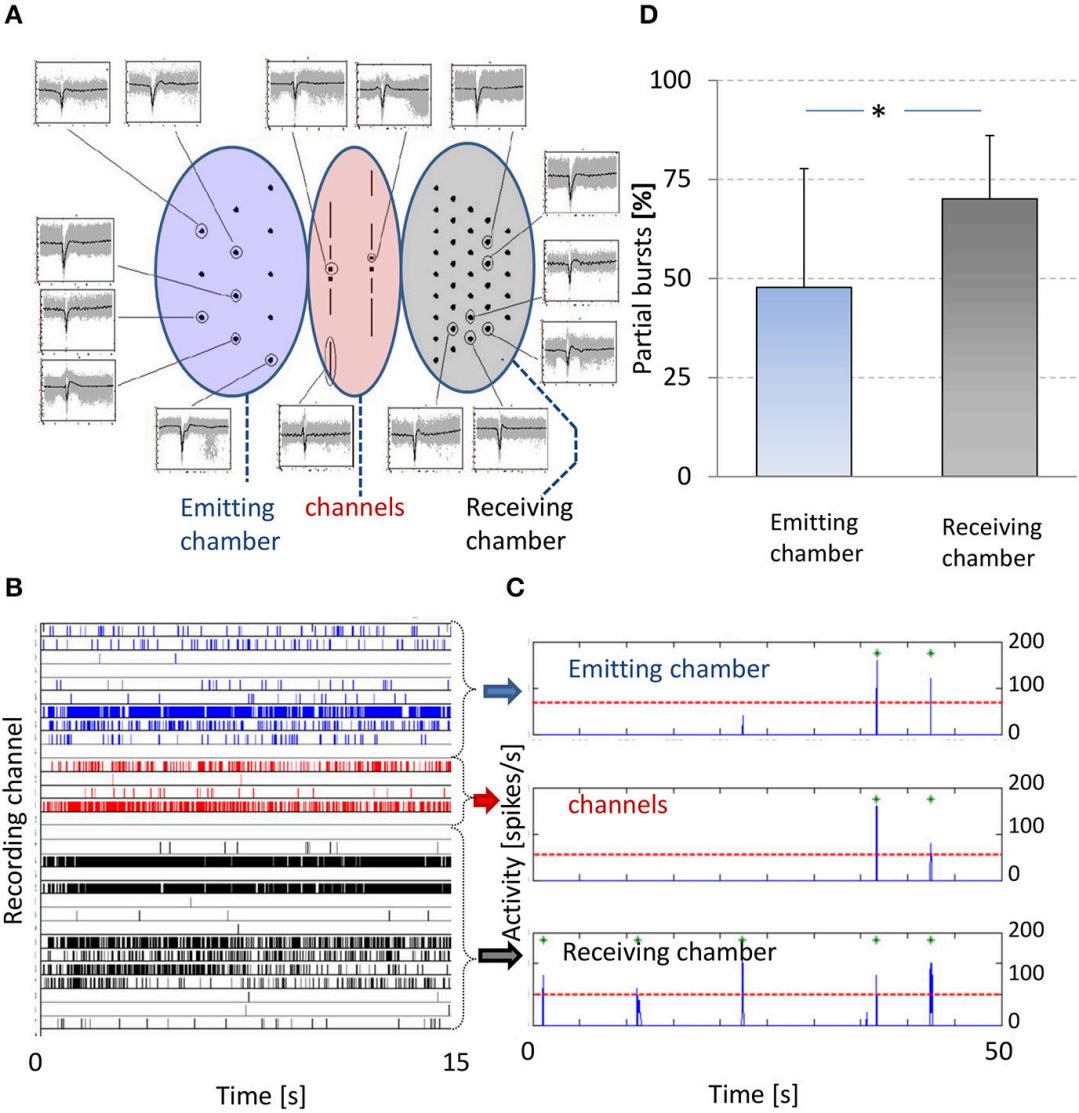
**Spontaneous activity**. **(A)** Center shows electrode layout and the periphery shows recorded action potentials. Left oval area (blue) indicates electrodes in the emitting chamber, middle oval area (red) indicates channel electrodes and right oval area (gray) indicates electrodes in the receiving chamber. Examples of individual action potentials (gray) are shown and their average shapes (black) per electrode, obtained from a 10 min recoding from a 3 weeks old culture. Only signals are shown of electrodes that recorded more than five spikes per minute. Horizontal axes span 6 ms, vertical axes are scaled to fit; mean amplitudes ranged 50–300 μV. **(B)** Raster plot of 15 s of recorded activity. Vertical axis indicates all recording electrodes, action potentials recorded by an electrode are indicated by tics in the corresponding row. All tics are color-coded to indicate the location of the recording electrode, blue (rows 1–10): receiving chamber, gray (rows 16–30): emitting chamber, and red (rows 11–15): channels. **(C)** Summed activity in 5 ms bins (blue line). A threshold (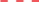) based burst detection algorithm found all bursts in either chamber and in the channels (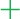). **(D)** Percentages of partial bursts in the emitting (blue) and receiving chamber (gray). Partial bursts were defined as any burst that occurred in one chamber at *t* = τ with no burst in the other chamber during the interval *t* = [τ−400; τ+400 ms]. The occurrence of partial bursts were expressed as percentages of the total number of bursts in either chamber. ^*^ paired *t*-test: *p* < 0.01.

In 14 experiments we were able to record spontaneous activity in both chambers and we calculated and cross correlated CWFR of both chambers. In 10 experiments (71%) we found a clear peak in the cross-correlation. Maximum cross-correlation was reached at a mean latency of 7 ± 19 ms (receiving chamber lagging), indicating a tendency that activity in the emitting chamber preceded activity in the receiving. In these experiments we also calculated the fraction of partial bursts (see Methods) in both chambers. In the emitting chamber the fraction of partial bursts was significantly lower than that in the receiving chamber (paired *t*-test: *p* < 0.01), see Figure 3D.

### Stimulus responses

**Figure 4:**
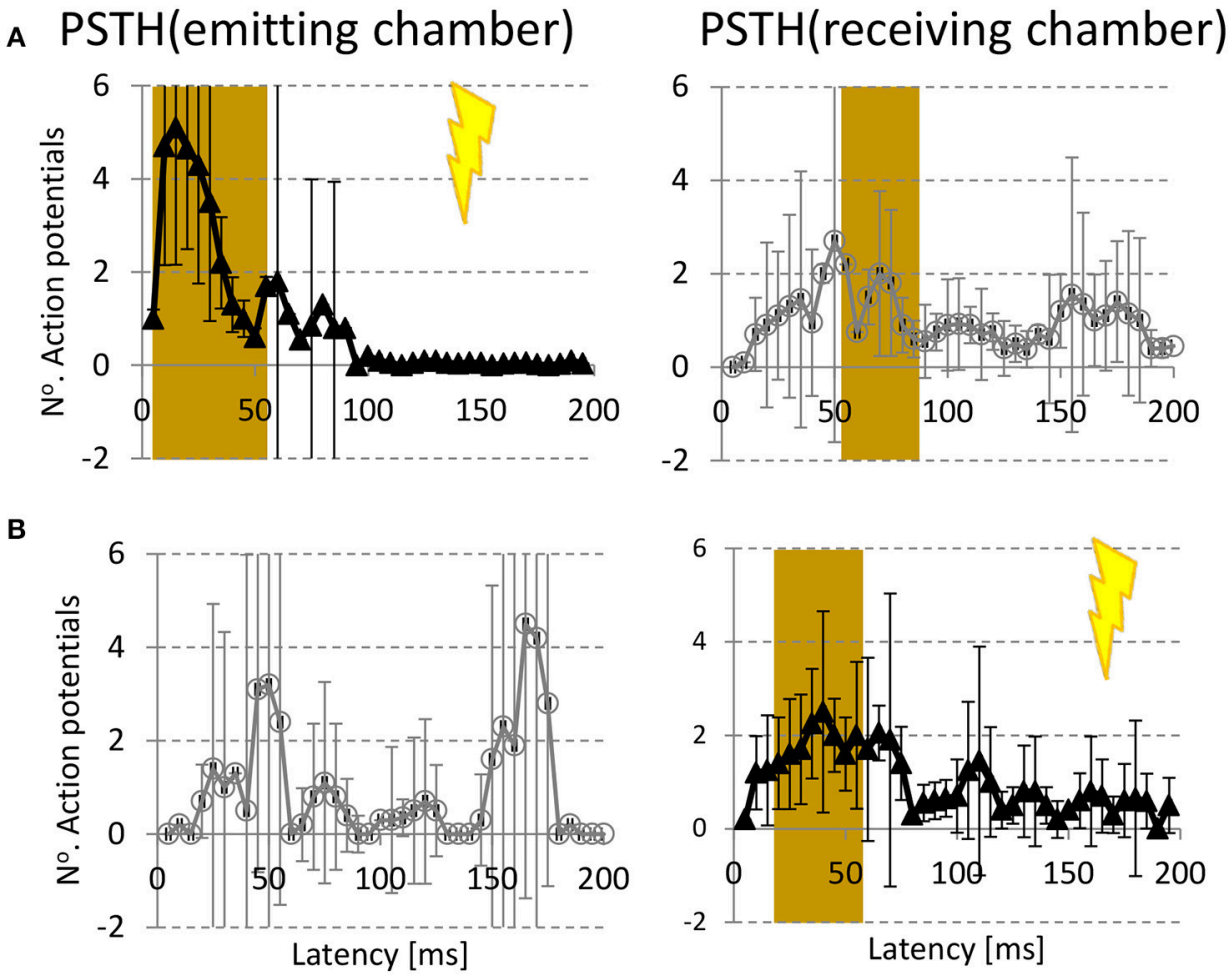
**Stimulus responses. (A)** Average post stimulus time histogram (PSTH) in emitting (left column) and receiving chamber (right column) to electrical stimulation in emitting chamber (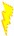) **(B)** PSTH(emitting chamber) and PSTH (receiving chamber) in response to stimulation in the receiving chamber (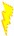). Error bars indicate 95% confidence intervals (= 1.96 × SEM), and represent differences between experiments (*n* = 12). Responses were considered significant if the 95% confidence interval did not include 0 in 2 consecutive time bins. Significant responses are indicated by brown backgrounds.

In 12 experiments from three different platings we sequentially stimulated all electrodes of both chambers in random order. We determined the mean PSTH per chamber in response to stimulation of all electrodes in the emitting (Figure 4A) or receiving chamber (Figure 4B). Stimulation in the emitting chamber yielded a significant response in that chamber at latencies in the range 0–55 ms, and a significant response in the receiving chamber at latencies 55–85 ms. Receiving chamber stimulation induced a significant response in that chamber at 15–55 ms, and no significant response in the emitting chamber.

## Discussion

Current experiments using cultured dissociated neurons on MEAs usually involve randomly connected, unstructured networks. However, structured networks may provide valuable new possibilities for experimenting. The activity patterns of cultured dissociated cortical neurons are usually dominated by network bursts. These bursts often hamper plasticity studies, and have been hypothesized to result from the lack of input to the cultures (le Feber et al., 2014). Electrical (Wagenaar et al., 2005) or pharmacological stimulation (Corner, 2008; le Feber et al., 2014) have been shown to reduce burstiness, and may thus facilitate plasticity studies. Here we developed a system where the main culture continuously received spontaneous or stimulated activity from an auxiliary culture. We used a newly designed DCMEA to achieve dual cultures that were (predominantly) coupled in one direction, that is, the culture in the emitting chamber provided input to the culture in the receiving chamber, but not vice versa. We validated the unidirectionality of the connections in several ways.

Visual inspection appeared difficult, and had the disadvantage that the direction of growth could no longer be determined after the axon had reached the other side of the channels. However, observations during growth show that axons initially grew through the channels from emitting to receiving chamber. It is possible that, once the first axons found their way through the channels, others followed their path, possibly also in the opposite direction. However, in a two compartment MEA with slightly lower tunnels, Pan et al. found that growth in the opposite direction was largely impeded because the tunnels were already filled (Pan et al., 2011). The applied staining to visualize growth inside the channels is not axon specific. Therefore, the observed neuronal processes in the channels in principle may have included dendrites. Lei et al. measured a total dendritic length of 723 ± 36 μm, distributed over 23 ± 1 branches of the dendritic tree on average (Lei et al., 2006), implying that the average dendrite length was far below the length of the 540 μm channels. Thus, it seems highly improbable that dendrites reached the other side of the channels, and therefore the stained processes were most likely axons.

Nine of the 20 channels contained two electrodes (see Figure 1), but it appeared quite difficult to record activity from both electrodes inside a channel. Possibly the design of the electrodes, some of which covered multiple (up to four) channels, introduced too much shunt to ground, if the electrodes were not sufficiently covered by axons in one or more of the channels. Nevertheless, we recorded activity from both electrodes in 14 channels. Cross-correlation indicated signal propagation from emitting to receiving chamber in 35% of those channels, and was indecisive in most other channels. Only the central channel had “private” electrodes, not shared with neighboring channels. Consequently, for most electrode pairs it was not certain that the recorded signals originated from the same channel. Even in those cases where activity at both electrodes did originate from the same channel, we could not verify that it was propagated by the same axons. It is however highly improbable that signals originating from different channels or from different axons in one channel consistently show a peak in their cross-correlation at sub-millisecond latencies. Recording from different channels or different axons may well explain the absent cross correlation peak in four of the 14 electrode pairs. Additionaly, the very small lag-time (0.12 ms, or, with f_s_ = 16 kHz, 2 samples), in combination with the much longer duration of an extracellularly recorded action potential [typically ≈1 ms (Gold et al., 2006)], possibly limited the precision of this approach. Still, the estimated mean propagation speed corresponded well with findings by Bakkum et al. (2013), although other studies (Dworak and Wheeler, 2009; Wieringa et al., 2010b; Brewer et al., 2013) reported slightly lower propagation velocities of 0.2–1.1 m/s, possibly reflecting the younger age of those cultures. Only one experiment suggested signal propagation in the opposite direction. Also the percentages of partial bursts were inverted in this particular experiment. Possibly, we erroneously rotated the PDMS top layer 180 degrees in this experiment. Because it was not possible to verify this after discovery, we nevertheless included the experiment, which increased standard deviations and negatively affected statistical power. This may have obscured a potentially even clearer demonstration of unidirectional connectivity.

CWFRs of both chambers included predominantly burst envelopes, and cross correlation indicated that bursts in the emitting chamber tended to precede bursts in the receiving chamber. In theory, it is possible that a burst started in one chamber, and that signals from a few axonal “burst-starters” then very rapidly propagated to the other chamber, evoking a burst there which grew mature even before the network burst became fully developed in the first chamber. Such a scenario might be the major cause of the relatively high standard deviation of the mean latency, and might in principle mask the actual direction of signal propagation. However, it seems very improbable that this happened in the majority of network bursts, and therefore the positive latency with maximum cross-correlation is presumed to be a good indicator of the direction of signal propagation and therefore of the direction of axon growth. The mean latency of 7 ms agrees well with values obtained by Renault et al. (2015). However, it is much larger than might be expected based on channel length and the estimated 2 m/s propagation velocity. This relatively large delay probably reflects the time needed to integrate enough EPSPs from the limited number of axons in the channels, to trigger a burst.

Also the lower fraction of partial bursts in the emitting chamber is indicative of better burst propagation from emitting to receiving chamber than in the opposite direction. In several cases of (nearly) simultaneously detected bursts in both chambers, it was not possible to determine in which chamber the activity was initiated. only partial bursts provide information on the channel polarity. Partial bursts are not propagated to the other chamber. Thus, the larger the fraction of partial bursts in a chamber, the lower the propagation probability. It is not clear how this selective functional connectivity relates to the number of axons inside the channels. Pan et al. estimated that a minimum of 100 axons are needed to reliably propagate bursts from one chamber to the other (Pan et al., 2015). Renault et al. argue that only a few axons would be necessary to relay a burst (Renault et al., 2015).

Responses to stimulation in either chamber further supported the unidirectional character of the channels. Stimulation of the emitting chamber elicited network responses in both chambers (with a longer latency in the receiving chamber), whereas network responses seemed restricted to the receiving chamber when the stimulus was applied there. The number of action potentials seems rather low at first sight, but it should be noted that these are measured per bin, and averaged across multiple stimulation electrodes, some inducing a stronger response than others. The average total number of spikes in response to emitting chamber stimulation equaled 29 and 11; responses to receiving chamber stimulation contained 0 and 14 spikes in the emitting and receiving chamber, respectively. Although we did not find a significant response in the emitting chamber, the curve of Figure 4B showed some peaks (around 50 and 170 ms), containing ~8 and 17 spikes on average. These peaks might result from spontaneous network bursts. Because it is improbable that spontaneous network bursts occurred multiple times at the same latency after the stimulus pulse, and Figure 4 shows averages of 10 stimulations at multiple electrodes. only extremely large bursts could have caused these peaks. It is therefore not plausible that these peaks were caused by spontaneous bursts. Alternatively, they might reflect occasionally present connectivity in the undesired direction, or antidromic activation of channel axons. The location of the reference electrode was chosen such that the electric field gradient was much larger inside the chambers than in the channels, when a chamber electrode was selected for stimulation. Still, it is possible that certain stimulation electrodes in the receiving chamber antidromically activated a sufficient number of channel axons to ignite a burst in the emitting chamber.

Although some measures, particularly visual inspection and cross correlation of signals recorded with two electrodes in a channel, appeared rather difficult or at the resolution limit, all presented evidence jointly supported the conclusion that on average the achieved connectivity between the two chambers was predominantly unidirectional.

An advantage of our approach is that we could already start recording from mature cultures after 19 days. However, Figure 2 shows that it took considerably longer than in Pan et al. (2011) for the axons to grow completely through the channels in the glass silicon DCMEAs, which largely reduced the gained time window for experimenting. Our channels were much longer than those in Pan et al. (2011) and contained 10 (glass-PDMS) to 20 (glass-silicon) barbs, which appeared to be far more than necessary to impede axon growth in the opposite direction. Electrophysiological data were obtained from glass-PDMS DCMEAs, with already reduced channel length and fewer barbs (10). Still, we may further improve the design by shortening the channels to 200–250 μm, containing 3–4 barbs. Adding a funnel shaped exit (Peyrin et al., 2011) at the receiving side might enable even shorter channels, but they should remain long enough to avoid that dendrites grow through. Alternatively, further improvement may come from the silicon DCMEAs, which offer superior visibility inside the channels and additional options for perfusion. Their failure to record activity so far, may be related to problems during the bonding process. In particular we found pollution of carbon and oxygen molecules on the electrode metal surface of the glass silicon DCMEAs, which suggests the high temperature during the anodic bonding procedure as a possible cause.

## Author contributions

JF designed the DCMEAs, analyzed the data and wrote the paper, WP did all experiments and analyzed the data, EW and MW designed and fabricated the DCMEAs, WR contributed to design of the DCMEAs, data analysis and writing of the paper.

### Conflict of interest statement

The authors declare that the research was conducted in the absence of any commercial or financial relationships that could be construed as a potential conflict of interest.
